# Study protocol for an evaluation of ASDetect - a Mobile application for the early detection of autism

**DOI:** 10.1186/s12887-019-1888-6

**Published:** 2020-01-18

**Authors:** Josephine Barbaro, Maya Yaari

**Affiliations:** 1 Olga Tennison Autism Research Centre, School of Psychology and Public Health. College of Science, Heath & Engineering. La Trobe University, Melbourne, Victoria 3086 Australia; 2 Goshen - Community Child Health and Well-Being. Haruv Campus for Children. Mount Scopus, 9765418 Jerusalem, Israel

**Keywords:** Autism, Early detection, Early identification, Developmental surveillance, Screening, E-health, Mobile application

## Abstract

**Background:**

Autism Spectrum Conditions (ASC) can be reliably diagnosed by 24 months of age. However, despite the well-known benefits of early intervention, there is still a research-practice gap in the timely identification of ASC, particularly in low-resourced settings. The Social Attention and Communication Surveillance (SACS) tool, which assesses behavioural markers of autism between 12 to 24 months of age, has been implemented in Maternal and Child Health (MCH) settings, with excellent psychometric properties. ASDetect is a free mobile application based on the SACS, which is designed to meet the need for an effective, evidence-based tool for parents, to learn about children’s early social-communication development and assess their child’s ‘likelihood’ for ASC.

**Study aims:**

The primary aim of this study is to evaluate the psychometric properties of ASDetect in the early detection of children with ASC. A secondary aim is to assess ASDetect’s acceptability and parental user experience with the application.

**Methods:**

Families are recruited to download the application and participate in the study via social media, health professionals (e.g., MCH nurses, paediatricians) and word of mouth. All participating caregivers complete a demographic questionnaire, survey regarding their user experience, and the Social Responsiveness Scale-2 (SRS-2), an autism screening questionnaire; they are also invited to participate in focus groups. Children identified at ‘high likelihood’ for ASC based on the ASDetect results, the SRS-2 or parental and/or professional concerns undergo a formal, gold-standard, diagnostic assessment. Receiver Operating Characteristic analyses will be used to assess psychometric properties of ASDetect. Thematic analyses will be used to explore themes arising in the focus groups to provide insights regarding user experiences with the app. Multiple regression analyses will be carried out to determine the extent to which demographic factors, parental stress and beliefs on health surveillance and child results on ASDetect are associated with the parental user-experience of the application.

**Discussion:**

With a strong evidence-base and global access, ASDetect has the potential to empower parents by providing them with knowledge of their child’s social-communication development, validating and reassuring any parental concerns, and supporting them in communicating with other health professionals, ultimately enhancing child and family outcomes and well-being.

## Background

### Autism early screening and diagnosis

According to the Diagnostic and Statistical Manual of Mental Disorders (DSM-5 [1]), Autism Spectrum Disorder (ASD) is a neurodevelopmental condition characterised by two broad areas of symptoms: 1) difficulties, differences, or deficits in social interaction and communication skills and, 2) the presence of restrictive, repetitive and/or sensory behaviours, interests and needs [1]. For the purposes of this paper, we will refer to ASD as “Autism Spectrum Conditions” (ASC) to reflect the broad autism spectrum, and heterogeneous support, needs, and strengths that autistic individuals and children on the autism spectrum represent [2–4].^1^ Current estimates of the prevalence of ASC are 1–2% of population [5, 6] {Bent, 2015 #13}. Whilst there has been advances in identifying biomarkers associated with ASC “likelihood” [7, 8], there are currently no reliable, universally applicable, biological markers to identify and diagnose ASC. Therefore identification of children at “high likelihood” for ASC can currently only be based on behavioural characteristics, via professional observations and parental report [9].

Early predictive behavioural markers of autism can be observed during the second year of life [10], and autism can be reliably diagnosed between 18 to 24 months of age [11–13]. However, the average age of diagnosis is still substantially later, at around 4 to 5 years of age [14, 15]. Consequently, many parents experience a “diagnostic odyssey”, with substantial delays and gaps between their initial concerns and their child’s diagnosis [16, 17]. The diagnostic gap is even greater for individuals living in socio-economic disadvantage, rural areas, and low-resourced settings [15, 18, 19], with prevalence rates of autism likely to be largely underreported in developing nations [20]. This research-practice gap in early identification of ASC prevents timely access to early intervention, which is known to enhance children’s cognitive, adaptive, and developmental outcomes, and inclusion in mainstream school settings [21–24]. Early intervention has also been found to reduce ongoing support for the child [23], family stress [25], and monetary costs associated with ASC across the lifespan [26].

The first potential barrier towards timely identification of autism is availability of feasible tools with good psychometric properties. The Social Attention and Communication Surveillance- (SACS, [27–29]) and the SACS-Revised (SACS-R, [30, 31]) tools, developed to address this need, assesses age-appropriate behavioural markers of autism for 11 to 30 month old children. Over the last 14 years, two large-scale community-based studies were conducted within the Victorian Maternal and Child Health (MCH) system in Australia. Over 400 MCH nurses were trained and ~ 38,000 children monitored with the SACS and SACS-R. The original SACS tool has excellent Positive Predictive Value (81%) for identifying ASC between 11 and 30 months of age, and excellent estimated sensitivity (84%), and specificity (99%) [27]); with similar psychometric properties found for the SACS-R (PPV: 83%; Negative Predictive Value – NPV; 98%; Specificity; 99.5%; Sensitivity; 77% [30, 31]). Overall, the SACS psychometric properties indicated that it is the most robust early detection tool for ASC for use in the general population, with evidence showing its efficacy in facilitating earlier diagnosis and intervention, leading to better cognitive outcomes, greater attendance at mainstream school, and less need for ongoing support [23].

As the SACS is best used within a universal healthcare system, whereby toddlers regularly have access to healthcare professionals trained to monitor children’s development, its use may be limited in communities where access to universal healthcare is absent or inadequate, or in developing nations where the resources to train professionals may be insufficient [18]. Moreover, there is increasing evidence that early parental concerns have predictive value in autism diagnosis; thus, structured parent report on early behavioural markers is valuable in enhancing the accuracy of early screening [32–34]. Another barrier for early identification of ASC is low parental awareness and knowledge of typical and atypical social-communicative development, especially among first-time parents [19, 35]. Finally, the knowledge of the early signs of autism amongst healthcare professionals has lagged behind the research, leading to many professionals adopting a ‘wait-and-see’ approach rather than taking immediate action upon presenting concerns by parents [36, 37]. This delay often leads to increased parental dissatisfaction and stress by shifting the responsibility back onto parents to seek answers to their concerns without the professional support they need. Parents whose concerns are validated by professionals feel supported and report higher satisfaction and reduced stress during the diagnostic process. Conversely, delays in the diagnostic process are consistently reported as a negative experience, associated with high parental distress and low satisfaction with the process [38–40].

A method whereby an ‘expert on the individual child’ (i.e., parent/caregiver) utilises evidence-based information, and taking this to ‘experts in children’s social communication development’, may prove fruitful in facilitating autism identification. Technology could therefore be used to bridge the gap that currently exists between parents’ first concerns and access to expert professional information in children’s social-communication development, providing them with evidence-based information to communicate with healthcare professionals and advocate for their child.

### ASDetect

**Fig. 1:**
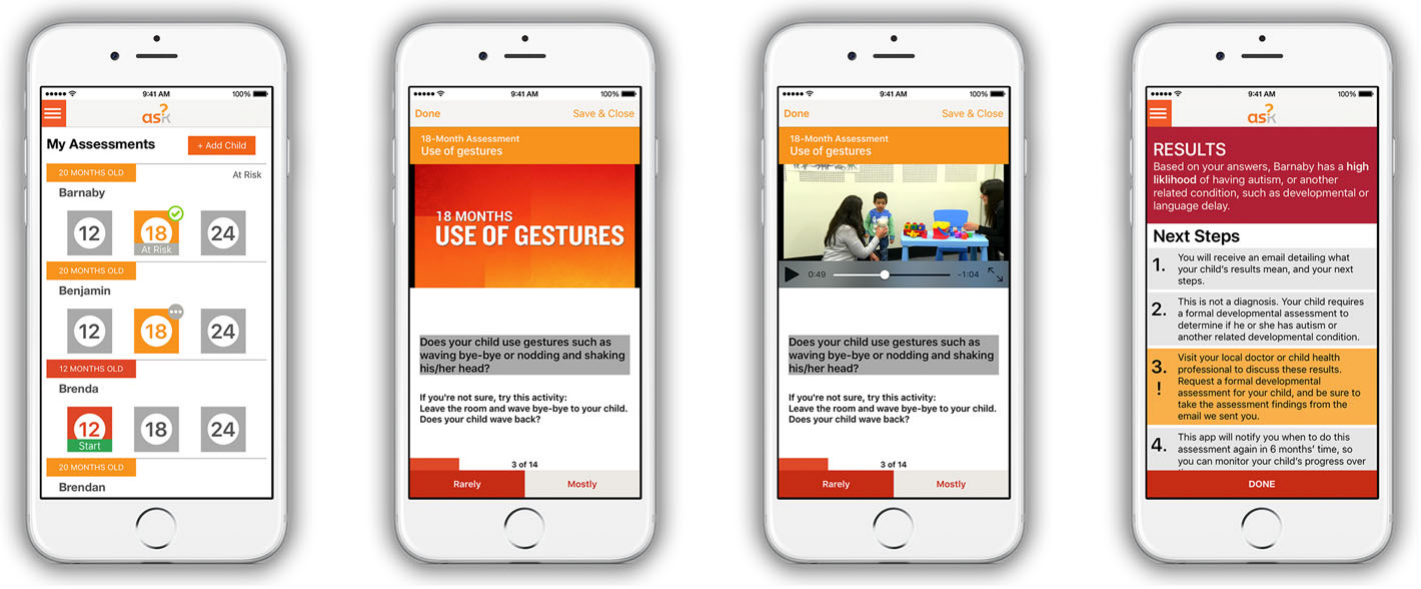
Example screenshots of the ASDetect application

ASDetect (asdetect.org) is a parent-led mobile application, available globally, free-of-charge, on Android and Apple platforms to anyone with smartphone or tablet access. It is designed to give parents of 11- to 30-month-old children the ability to assess their child’s ‘likelihood’ for autism in their own home, based on the early behavioural markers of autism identified in the SACS [27–29]. With more than 5 billion people connected to mobile services in 2017, and an expected increase of a billion new mobile uses over the next five years, mostly from developing countries (80%), mobile apps have become highly accessible, with greater global reach than any other technology [41]. By translating the SACS behavioural items into an app, ASDetect was designed to address barriers associated with accessing timely and expert advice on children’s social-communication milestones, thereby bridging the research-practice gap in the early identification of autism. See Fig. 1 for example screenshots of ASDetect.

The SACS behavioural items incorporated into ASDetect are accompanied by narrated videos by the first author (JB), demonstrating key social-communication behaviours at 12, 18, and 24 months of age, comparing autistic, and non-autistic, children; this resulted in an app that is both an early detection *and* education tool for parents. Behaviours found to be most predictive of autism in the SACS [27, 28] are used in the app to determine a child’s ‘likelihood’ for autism (high/low). Parents who are concerned about their child’s development are encouraged to share these empirically-based results with their doctor, in an attempt to reduce the barriers associated with a ‘wait-and-see’ approach. Since released in February 2016, ASDetect has been downloaded over 44,000 times globally.

### Study aims

The primary aim of this evaluation study is to assess the psychometric properties of ASDetect in identifying young children with ASC. A secondary aim is to describe parental/caregiver acceptability, satisfaction, and user-experience of ASDetect and how they are associated with parental/caregiver demographics, stress, and beliefs regarding health and developmental screening, as well as child characteristics and results on the ASDetect.

### Methods

#### Study design

**Fig. 2:**
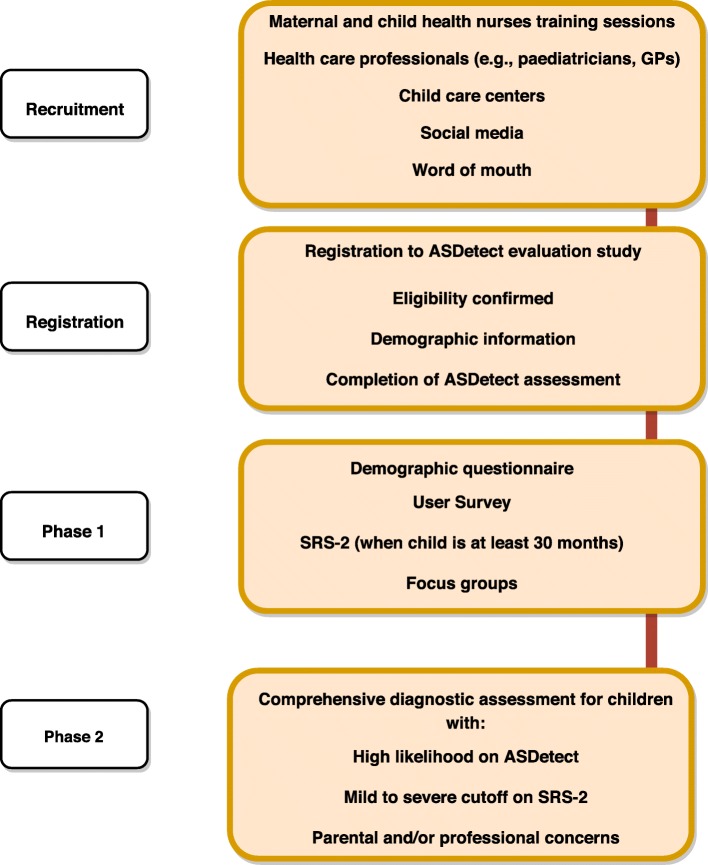
Study design

The study includes two phases: Phase 1 involves all families who have registered for the ASDetect Evaluation Study and complete at least one assessment for their child in ASDetect; they then complete a survey regarding their user-experience, and an autism screening questionnaire (the Social Responsiveness Scale (SRS-2, [42]). All parents are also given the opportunity to participate in focus groups. Phase 2 involves a formal assessment for: 1) children at “high likelihood” for autism based on the ASDetect results; 2) children who meet the mild to severe SRS-2 cut-off for autism; or 3) parents and/or professionals who have concerns about a child’s likelihood for autism (see Fig. 2). Inclusion criteria for participation are: family resides in Australia; the child is between the ages of 11 and 30 months; parents have a mobile or tablet device compatible to run ASDetect; and parents have an active email address. To be representative of general community samples, no exclusion criteria based on preterm birth, neurological, genetic, or other medical conditions are applied.

#### Recruitment

Families are invited to use ASDetect via advertising through social media (i.e., Facebook, Twitter), word-of-mouth, and pamphlets delivered to health, community, and education centres (i.e., General Practitioners, Early Childhood Educators, and Allied Health Professionals). Another stream of recruitment is practitioner-led recruitment via Maternal Child Health (MCH) nurses (see Additional file 1 – recruitment flyers). In the State of Victoria, Australia, infant and child development is monitored through the universal MCH service, which is offered free of charge to all families with children up to 3.5 years of age. One of the aims of this service is to monitor children’s growth and development, with well-baby checks scheduled at key ages from birth to 3½ years. Information regarding participation in the study was presented at a monthly MCH coordinators meeting, with informed consent for their local councils given on the day, or via a follow-up email. Following this, all MCH nurses in participating councils attend training sessions, where they are provided information by the research team about early identification of ASC, ASDetect, and the study methodology; they are also coached on how to support parents who have concerns about their child’s development.

### Procedure

#### Phase 1

This phase involves all registered parents and is designed to assess parental user experience and satisfaction with ASDetect, and the process of assessing their child’s social-communication and “likelihood” for ASC. The recruitment flyers provide information to text “app” to a specified mobile number that takes parents to the evaluation study registration website, or by entering the website address into their computer/mobile internet browser. The evaluation study website contains information about the study and an Information Statement and Consent form, followed by a registration form to determine eligibility to participate and collect basic demographic information (i.e., contact details, council of residence, parental age and gender, child age and gender, parental concerns, how they heard about the app, and whether the participating child has siblings diagnosed with ASC). All families registered in the study are instructed to download and use ASDetect. Upon completion of at least one ASDetect assessment, all parents are prompted to complete a demographic questionnaire and a user experience survey, and invited to participate in focus groups.

#### Phase 2

This phase is designed to assess the psychometric properties of ASDetect in identifying children on the autism spectrum. This phase involves a free developmental assessment by the ASDetect team at La Trobe University (LTU) for children at ‘high likelihood’ for ASC on ASDetect. When recruiting for participation in the study, families are notified that they may be contacted by a researcher at some stage during the study and invited to participate in research activities, but no information about what this entails is given. This is to ensure that participants do not actively seek to produce a ‘high likelihood’ result on ASDetect to access the free assessment.

**Fig. 3:**
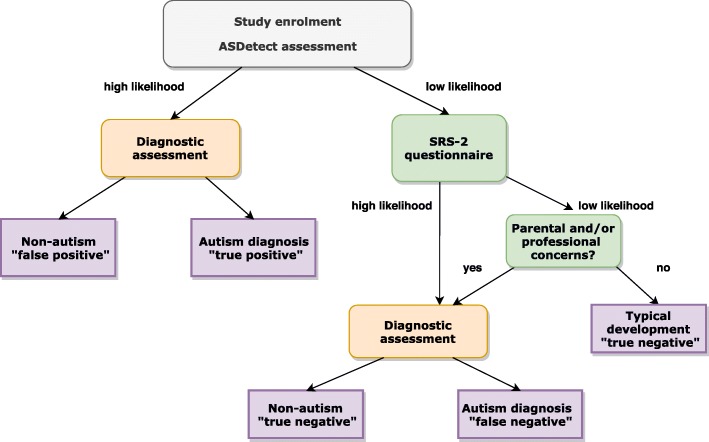
Flow chart of decision making for assessment protocol and outcomes

Additionally, parents of *all* children (both high and low likelihood) are sent, via email, the SRS-2 to fill out when the child is at least 30 months of age. Children with a “low likelihood” result on ASDetect but score in the “mild to severe” range for autism on the SRS-2, are invited for an assessment. Having SRS-2 data for all children allows us to use established cut-offs for autism on the SRS-2 to estimate false negatives rates in the “low likelihood” group. Additionally, parents/caregivers, MCH nurses, or other professionals who contact us with concerns about a child’s development (regardless of the concern) are also invited for an assessment (see Fig. 3).

Following the in-person assessment at LTU, parents/caregivers are counselled about their child’s assessment results, referred to appropriate services and resources, and provided with a comprehensive developmental and diagnostic report. Children who initially undergo an assessment at 12 months are invited back for a follow-up assessment at 18 and 24 months, and children seen at 18 months are invited for a follow-up assessment at 24 months, with a final “Best Estimate Diagnosis” based on all clinical information made by the clinical team. Children seen initially at 24 or 30 months are seen only once, except in circumstances where a diagnostic decision was not able to be made at this time, and the family is invited back once more in 6 months’ time for a final clinical assessment and diagnostic decision.

Over and above the procedure specified above, developmental assessments to find any further “false negatives” for children with a “low likelihood” ASDetect result, *and* no parental or professional concerns (of any kind, not just ASC), *and* scoring below the autism cut-off on SRS-2, are not conducted, as it is not ethically, practically, or financially feasible to do so.

### Measures/tools

#### Phase 1

***ASDetect App (******asdetect.org****)* **-** After downloading the app and providing basic details, the most relevant assessment is then selected for the parent to complete, with three age-windows available (12-months: age range 11–15 months; 18-months: age range 16–21 months; 24-months: 22–30 months); participants are unable to complete an assessment that is not within the age parameters of the child being assessed. Each assessment contains 10–15 behavioural items, with five “key” markers of autism at each age, which are a direct translation of the SACS-R items, as identified in [28]. For each behavioural item, parents are required to respond whether their child completes a behaviour ‘rarely’ (an “atypical” behavioural response) or ‘mostly’ (a “typical” behavioural response), which corresponds to the SACS-R items. Most of the items are accompanied by a video and/or brief activity for the parent to complete with their child, to give an indication of whether their child is, or is not, performing the behaviour in question. Videos contain typically developing children and children on the autism spectrum engaging or not engaging in the behaviour, while the narrator (JB) highlights key learnings throughout the videos, often with a pause on the behaviour (e.g., an instance of eye contact or “joint attention”). Assessments take approximately 20 min to complete, and parents can stop and resume the assessment, as required, as long as the child is within the age window.

Email reminders are sent after seven days if a family has registered a child but has not completed the relevant assessment, and another reminder is sent 28 days before the eligibility window expires for their child’s assessment. ASDetect sends participants reminders that their child’s next assessment can be completed, until this no longer becomes relevant (i.e., the 24-month assessment is complete, or child is over 30 months old). This is to encourage a “developmental surveillance” approach to the early identification of autism, upon which the SACS-R framework is based.

Upon completion of the assessment, users are given an immediate on-screen result of either ‘low’ or ‘high’ likelihood of autism for their child, accompanied by brief “next steps” (e.g., see their local doctor to request a formal assessment for autism). Users also simultaneously receive a comprehensive formal assessment results email summarising their child’s results, whether the child was identified as performing each of the behavioural items ‘typically’ or ‘atypically’, as well as a summary and link to the evidence on which ASDetect is based. This email is designed to be taken to their local doctor or other relevant healthcare professional.

***Demographic Questionnaire*** – a demographic questionnaire is completed by the child’s primary caregiver, detailing each caregivers’ age, education, occupation, employment status, and country of birth; child’s age, gender, and place of birth; other languages spoken at home; family structure and annual income; number of siblings and any family history of autism or other developmental conditions.

***The ASDetect Feedback and Outcome Questionnaire*** is a purpose-developed, self-report questionnaire administered to the parents/caregivers via email one month after completion of the first ASDetect assessment. The questionnaire was constructed using some items from Crane et al.’s (2016) survey [38], adapted to reflect the use of a screening and developmental surveillance app for autism. Prior to its use, this survey was reviewed by a panel consisting of three experts in the early presentation of autism, and three parents whose child received a diagnosis of ASC. The purpose of this review was to ensure the survey was readily understandable and easy to complete, while at the same time being as comprehensive as possible. The survey was divided into several sections, as described below**:**

#### The diagnostic process

Parents/caregivers are asked to indicate the age of the child when initial concerns were noted, when these concerns were first raised with a health professional, and what the outcome of the consultation was. Parents/caregivers are also required to detail any subsequent outcomes after receiving an on-screen result of either ‘low’ or ‘high’ likelihood of ASC from the ASDetect app (e.g. visits to any health professionals and feedback/outcomes received from these visits).

#### Satisfaction with ASDetect

Using 5-point Likert scales (‘strongly agree’ to ‘strongly disagree’), parents/caregivers indicate their satisfaction with different aspects of the app, as well as their overall experience in using ASDetect as an early screening and developmental surveillance tool designed to assess their child’s likelihood of autism.

#### Disclosure of results through ASDetect

Using 5-point Likert scales (‘strongly agree’ to ‘strongly disagree’), parents/caregivers report on the process of receiving results from ASDetect regarding their child’s likelihood of ASC, whether they would have preferred to receive the results in the presence of a primary health care professional, and their feelings/emotions at the time.

#### Beliefs regarding health screening and developmental surveillance

Using 5-point Likert scales (‘strongly agree’ to ‘strongly disagree’), parents/caregivers report on their beliefs relating to general health screening and autism-specific developmental surveillance.

#### Parental stress

The Kessler Psychological Distress Scale (K10 [43];), is included to assess parent/caregiver level of distress in the 30 days after receiving results from an ASDetect assessment**.** The K10 is a widely used screening measure to assess psychological distress in clinical and epidemiological research, with norms validated in the Australian population [44]. Items are scored from 1 (none of the time) to 5 (all the time), with higher scores indicting higher levels of distress.

***The Social Responsiveness Scale–Second Edition (SRS-2, [42]***) is scale measuring differences in social behaviour associated with ASC. It is completed by caregivers of children aged 2.5 to 4.5 years, and includes 65 items scored on a 4-point Likert-type scale. T-scores (*M* = 50, *SD* = 10) are obtained for five subscales and the overall total score: Social Awareness, Social Cognition, Social Communication, Social Motivation, and Restricted Interests and Repetitive Behaviour. Age-normed cut-offs for total T-scores are available to indicate the presence and severity of clinically significant difficulties in social functioning that interfere with interactions with others. The SRS-2 has excellent predictive validity for autism, with sensitivity of .92 and specificity of .92, based on a general population standardisation sample [45].

***Focus groups*** – all parents registered in the study are invited to attend focus groups at LTU, and participants are compensated for their time and travel costs. The focus groups are conducted separately for parents of children with “high” and “low” likelihood results on the app. Parents are prompted to discuss their views and experience of using the app, and how this affected the diagnostic process (when applicable), their stress, and coping. A moderator guide for these groups was developed in consultation with two experts in focus groups and qualitative research, who also reviewed the guide. Additionally, it was reviewed by three experts in autism who also have experience in conducting or supervising focus groups. The groups take place at a secure, private room, and are facilitated by a researcher who was not involved in the development of ASDetect. The sessions are recorded, and then transcribed by two researchers. The NVivo10 software [46], will be used to analyse the data, and analysis will be a conducted by two independent researchers specialising in qualitative research.

### Phase 2 - gold standard diagnostic assessment

The developmental assessment includes gold-standard tools for early autism diagnosis (detailed below), based on the child’s age. The children are assessed by a research team of registered psychologists who are researcher-reliable on the assessment measures, supervised by the lead author (JB), who has extensive expertise in the early autism phenotype. The clinicians are blind to the child’s ASDetect results, to decrease potential biases in the initial assessment outcomes. Based on the standardised observational measures, clinical observations, and parental reports, clinicians assign the child a best estimate diagnostic status at 12 and 18 months, and diagnosis is confirmed at 24 months, or at 30/36 months, if required, based on the child’s presentation.

***Mullen Scales of Early Learning (MSEL*)** - The MSEL [47] assess developmental functioning of children from birth through 68 months of age. The Early Learning Composite score offers a standardized general score (*M* = 100, *SD* = 15) based on four standardised scales (*M* = 50, *SD* = 10): fine motor, visual reception, expressive language, and receptive language. The MSEL is widely used with infants and toddlers in ASC research [48, 49]. Excellent test-retest and inter-rater reliability of the scales were reported for ages ≤24 months (r ≥ .82), as was congruent validity with other measures [47, 50].

***Autism Diagnostic Observation Schedule-2 (ADOS-2*) –** the ADOS [51] is a semi- structured, standardised, observational assessment designed to assess behaviours related to ASC. It provides several opportunities for communication, social interaction, and play or imaginative use of materials, and it measures social and communicative behaviours diagnostic of autism. A subset of items comprises the diagnostic algorithm of the ADOS-2, structured in two domains: Social Affect and Restricted Repetitive Behaviours. An algorithm score is calculated, indicating whether the child meets diagnostic criteria for autism. A Toddler Module (ADOS-T [52];) was designed for use with children aged ≤30 months. For this module, due to the child’s young age, algorithm scores indicate three levels of concern (little-or-no concern, mild-to-moderate concern, moderate-to-severe concern), rather than diagnostic “cut-offs”. Excellent test-retest and inter-rater agreement (Intra-class correlations ≥ .90) and excellent sensitivity & specificity for identifying ASD versus other developmental conditions (≥. 81) were demonstrated.

***Autism Diagnostic Interview-Revised (ADI-R,*** [53] is a structured interview used for assessment of individuals suspected of being on the autism spectrum. A clinical interviewer questions a parent or caregiver regarding the child’s developmental history and current behaviour and is suitable for children aged 24 months and above. The interview includes open questions about child’s history and parent concerns, communication behaviours, social development and play skills, repetitive and restricted behaviours, and questions about other behavioural difficulties. The ADI-R has diagnostic algorithm scores, providing categorical results indicating whether the symptoms reported meet criteria for an autism diagnosis. It has shown excellent test-retest and inter-rater agreement (Intra-class correlations ≥ .92) and excellent discriminant validity between ASD and non-ASD for each of the domains (*p* < .0001).

***Developmental Interview*** – a semi-structured developmental interview, developed by the lead author (JB), is administered during the assessments of children who are < 24 months of age. Caregivers are asked about the child’s medical and developmental history, child’s attainment of developmental milestones and social-communication skills, the presence of any restricted, repetitive, or sensory behaviours or interests, their current skills and difficulties, and parental concerns.

***Vineland Adaptive Behaviour Scales-3 (VABS-3)*** – The Vineland-3 [54] is a commonly used measure to assess children's daily living skills in four domains (communication, daily living skills, socialisation, and motor skills), as well as a “Maladaptive Behaviors” domain. The caregiver form, which takes 10 min to complete, is used in the study. Caregivers are asked to rate whether the child currently exhibits each described behaviour or not. The four domain scores are standardised and compose the Adaptive Behaviour Composite Score (*M* = 100, *SD* = 15 in domain and composite scores). The Vineland-3 has strong psychometric properties, with internal consistency alpha coefficients ranging from 0.90 to 0.98, test-retest reliability coefficients ranging from 0.80 to 0.92, and inter-rater coefficients from 0.70 to 0.79.

***Brief Infant Sleep Questionnaire (BISQ***, [55]) – the BISQ is a short screening questionnaire for infant sleeping problems. It is designed for infants aged ≤29 months and includes 13 questions regarding three domains: Nocturnal sleep duration, night waking, and methods of falling asleep. The BISQ has high test-retest reliability (0.81 to 0.95) and has been validated against actigraphy and daily logs [55]. Parents filled-out this questionnaire for children who were 24 months or younger.

***Child Sleep Habits Questionnaire (CSHQ***, [56]) -the CSHQ is 45-item questionnaire assessing sleep behaviour in young children. It includes items relating to bedtime behaviour and sleep onset; sleep duration; anxiety around sleep; behaviour occurring during sleep and night waking; sleep-disordered breathing; parasomnias; and morning waking/daytime sleepiness. The CHSQ has strong psychometric properties for use in community and clinical samples, with internal consistency coefficients ranging from 0.38 0.93 and test-retest reliability ranging from 0.62 to 0.79 [56]. Parents filled this questionnaire for children who were older than 24 months.

***Behaviour Assessment System for Children (BASC-3,*** [57]). The BASC-3 is s a well-established comprehensive measure of a child’s adaptive and problem behaviours. It contains 139–175 items and yields a total score. Items include a wide array of behaviours that represent both behavioural problems and strengths, including internalising problems, externalising problems, school problems, and adaptive skills. Reliability coefficients for composite score of the parent ranges from .93 to .97, and test-retest reliability coefficients from .88 to .92 [57]. Parents filled this questionnaire for children who 24 months and older.

### Data management

Data gathered by the app is collected in two places. The data entered by parents is transferred to a PostgreSQL database sitting on Heroku. Heroku is a platform upon which the app was built, and any information sent to or from ASDetect comes through Heroku. Information collected by Heroku is only accessible to the technical support team and is coded. Data is then transferred and stored in Salesforce. Salesforce is a secure, web-based, password-protected Customer Relationship Management (CRM) platform. All collected data that is available for use will be accessed and exported by authorised personnel in the research team via Salesforce.

Caregiver questionnaires (detailed above) are completed via Qualtrics [58], and the data is stored in an electronic database that is only accessible to study researchers. For families who request to complete the "paper-based" questionnaires (as opposed to online on Qualtrics) these are sent to the families prior to, and collected at, their visit. Information provided, as well as the assessment results, are transferred onto the electronic database that is only accessible to study researchers.

### Analytic plan

#### Sample size

A Receiver Operating Characteristic (ROC) power analysis using MedCalc (alpha = .05, beta = .20 [1-Power], Area Under the Curve = .8 [good-excellent], a conservative ratio of negative/positive cases for autism = 99/1), revealed that the minimum number of children needed to be monitored with ASDetect is 700 for .80 power. We therefore aim to monitor a total of 1000 children altogether to allow sufficient power and account for drop-out and consent to participate. Based on pilot results from completed ASDetect assessments, approximately 18% of children monitored were identified at ‘high likelihood’ for autism; this is a much higher rate than that found in the SACS-R (~ 2%), which may be explained by the fact that 71% of parents indicated in a previous user survey (*N* = 122) that they had prior concerns regarding their child’s development prior to using the app [59]. Thus, an estimated sample of 180 children will be identified at ‘high likelihood’ for ASC on ASDetect and invited for a gold-standard developmental assessment. Pilot data indicate a false positive rate of 16% for ASC (Positive Predictive Value: 84%), with these children having developmental/language delays (i.e., not typically developing). Thus, we anticipate that approximately 29 children with a “high likelihood” result on ASDetect will not have ASC; these children are likely to have language/developmental delays or other conditions. We do not have sufficient previous data to estimate the number of additional children to be invited for assessment based on SRS-2 results or parental and/or professional concerns and their anticipated outcomes.

#### Quantitative analyses

Aim 1: Psychometric properties of ASDetect - Based on the outcomes following children’s diagnostic follow-up assessments and the results on the SRS-2, four groups of children will be identified: 1) “true positive” – children who are identified at “high likelihood” for autism on ASDetect and their diagnosis is confirmed as ASC; 2) “false positive” – children who are identified at “high likelihood” for ASC on ASDetect and their diagnosis was not ASC (although we still anticipate these children will present with clinically significant difficulties and delays other than ASC, based on current pilot data; thus, we do not anticipate “true” false positives of children presenting with typical development); 3) “false negative” - children who are identified at “low likelihood” for autism on ASDetect and have received a diagnosis of autism – these children will be identified via: a) scoring above the SRS-2 cut-off for ASC, b) parents/professionals have contacted us with their concerns about the child, c) incidental reports by parents to the research team informing us that the child received a diagnosis in the community by a paediatrician, registered psychologist, or multidisciplinary team; and 4) “true negative” - children who are identified at “low likelihood” for autism on ASDetect and have not met any of the criteria as mentioned in point three above. Sensitivity, specificity, positive and negative predictive value will be estimated based on these rates using ROC analyses. The validated SRS-2 cut-offs will be used and their concordance with the results of the gold standard assessments will be examined.

Aim 2: Acceptability and user experience - Survey responses from all families will be analysed and descriptive results will be used to assess the parental user experience of the diagnostic process using ASDetect, their satisfaction, and their opinions regarding disclosure of the results via the app. The survey will also inform referral pathways leading families to the app (e.g. MCH nurse, social media), and potential enablers and barriers to using the app (e.g., parental education, geographic location, having another child on the spectrum). Factor analysis will then be used to generate overall acceptability and user experience scores based on survey responses. Multiple regression analyses will then be carried out with these scores as dependent variables to determine the extent to which demographic factors, parental stress, beliefs on health surveillance, and the assessment results are associated with their user-experience with ASDetect.

#### Qualitative analyses

In addition to the quantitative data regarding acceptability collected via user surveys from all participants, two focus groups (“high” and “low” likelihood groups) will be conducted to collect in-depth information regarding users’ experiences, including their views, beliefs, and motivations in using the app. Raw data from the focus groups will be coded, and the data will be categorised into key themes by two independent researchers. Two coders will independently code the transcripts to establish interrater reliability of at least 80% agreement [60]. To best fit the data, and reduce overlap and redundancy, themes will be added or removed. Finally, a framework will be constructed based on the major themes and processes identified during coding [61, 62].

## Discussion

The main aim of this study is to evaluate the psychometric properties of ASDetect, an evidence-based app for the early detection of autism. This will be investigated through Receiver Operating Characteristic analyses, based on diagnostic outcomes of children identified at ‘high likelihood’ for ASC based on ASDetect results, the SRS-2 or parental and/or professional concerns. A secondary aim is to describe and predict acceptability, satisfaction, and user-experience of ASDetect with parents and caregivers.

One of the strengths of this evaluation study is its use of a community-based sample. Many studies assessing early autism “likelihood” use selective “high-risk” samples (e.g. siblings of children diagnosed with ASC or clinically-based samples), and are more likely to include children with less symptomatic autism, and families with higher socio-economic background, limiting the generalisability of the results [63]. Based on a community-based sample, this study may be more representative and applicable to the general population of children on the autism spectrum.

The main limitation of this large-scale study is that it is not ethically, practically, or financially feasible to assess all 1000 children who are registered as part of the study, who: 1) were not at ‘high likelihood’ for autism, *and* 2) did not have any parental and/or professional concerns, *and* 3) did not score above the autism cut-off on the SRS-2. This is an (unfortunately) necessary limitation of large-scale screening studies, which means that *true* “false negatives” can only be estimated based on random sampling of a subset of children at “low likelihood” of autism, or, as per this study, follow-up of ALL children who have parental and/or professional concerns (of any kind, not just autism), and inclusion of a secondary screener (SRS-2), implemented when the children are older (at 30 months).

In conclusion, ASDetect, an evidence-based app for the early detection of autism, provides a “safe space” for parents to explore their concerns in their own home, prior to raising these concerns with a professional and/or other family members, which can often act as a barrier to help-seeking. It has the potential to empower parents in communicating their concerns and support them in engaging their local, relevant, healthcare professionals. It also can promote parent literacy on social-communication milestones and the early indicators of autism – thus, ASDetect is both an early detection *and* education tool for autism. Furthermore, by 2020, 80% of the world’s 6 billion smartphone users will be from the developing world, providing enormous scope for ASDetect’s use in these countries [18].

## Supplementary information

**Additional file 1.** 21576 ASDetect Eval Study DL_Brochure

## Data Availability

Data will be available upon request.

## Abbreviations

ADOS: Autism diagnostic observation schedule
ASC: Autism spectrum conditions
ASD: Autism spectrum disorder
SACS: Social attention and communication surveillance
SRS-2: Social responsiveness scale-2
MCH: Maternal and child health
MSEL: Mullen scales of early learning
ADI-R: Autism diagnostic interview, revised

## Footnotes

1 Identity-first language (e.g. autistic individuals) is the preferred language of many autistic people and their parents (3–4). However, as there is no universally accepted way of describing autism (4) the preferences of “person-first” language amongst individuals and parents in the autism community is also acknowledged in this paper. Thus, the terms “autistic individuals” and “children on the spectrum” will be used in this paper to reflect the autism community’s spectrum of preferences in terminology.
