# Supplementary material for: Study protocol for an evaluation of ASDetect - a Mobile application for the early detection of autism
Source: BMC Pediatr. 2020 Jan 18;20:21. doi: 10.1186/s12887-019-1888-6 (PMC6969425; doi:10.1186/s12887-019-1888-6)
Supplement: Supplementary file 1 — Additional file 1. 21576 ASDetect Eval Study DL_Brochure [file 12887_2019_1888_MOESM1_ESM.pdf]

WHY IS POINTING SO IMPORTANT?

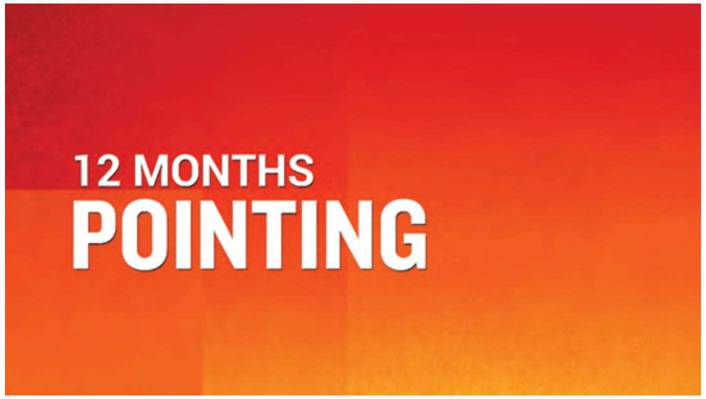

Pointing and other skills like eye contact and responding to name are important early developmental milestones. Their absence can be associated with Autism, but discovering this early gives the best possible chance for every child to fulfil their potential. This is why early identification is critical.

ASDetect allows you to monitor your child's social and communication skills through a combination of short videos, questions, and activities you can do with your child.

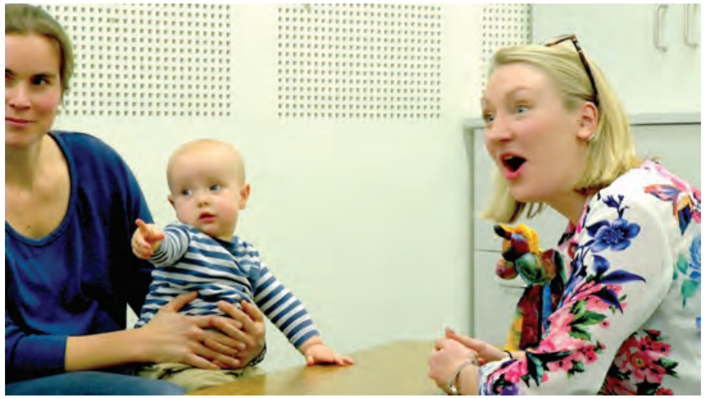

Help us improve our world-first early autism detection app

We're currently seeking parents/caregivers of young children for a new study, looking at how our mobile app ASDetect identifies young children showing early signs of Autism.

You can participate if:

- You have at least one child aged between 11 and 30 months old
- You have an active email account
- You have a mobile phone or tablet (Android or iOS).

Olga Tennison AUTISM RESEARCH CENTRE AUSTRALIA

Researchers
Olga Tennison Autism Research Centre
School of Psychology and Public Health
La Trobe University

Dr Josephine Barbaro
Prof Cheryl Dissanayake

03 9479 1283
appstudy@asdetect.org

This study is funded by a La Trobe University Research Focus Area grant and has been approved by La Trobe University's Human Ethics Committee No. HEC 17-022.

LA TROBE UNIVERSITY | 50 YEARS

ASDetect

Do you have a child aged between 11 and 30 months?

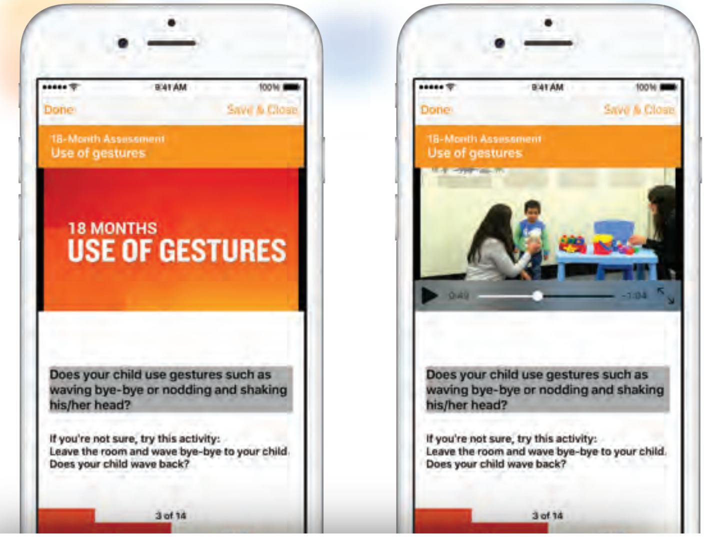

Learn why pointing and other communication milestones are important for your child's development by joining a new study.

SMS **"app"** to **0409 758 602**
or visit
asdetect.org/app

WHAT IS INVOLVED?

You'll use our free app ASDetect to monitor your child's social attention and communication behaviours. Each assessment takes approximately 20 minutes, followed by an instant on-screen result and follow-up email.

Depending on how old your child is, you might be asked to complete another one or two in-app assessments at 6 and/or 12 months after the first.

A research team member may also invite you to participate in the second voluntary phase of the study.

1 REGISTER

You MUST register by visiting **asdetect.org/app** or SMS **"app"** to **0409 758 602**.

Simply downloading and using the app will NOT register you for the study.

2 ADD CHILD

After registering yourself online, you'll add your child.

3 SET USER NAME AND PASSWORD

You'll then receive an email with your consent form and study information.

.

4 DOWNLOAD ASDETECT

GET IT ON Google Play | Download on the App Store

Available on Google Play or the Apple App Store.

.

5 COMPLETE ASSESSMENT

Open ASDetect on your device, and enter your user name and password. Your child will appear and you can then do an assessment. Assessments take approximately 20 minutes, including explanatory videos.

6 VIEW RESULTS

You can review your answers before submitting an assessment. You'll receive an instant result of 'higher' or 'lower' likelihood of autism, and a follow-up email.

NEXT STEPS

After you receive your child's result, you may be contacted to participate in the second voluntary phase of the study. Regardless of your result, if you have any concerns please contact us and see your GP or paediatrician.

LA TROBE UNIVERSITY

ASDetect

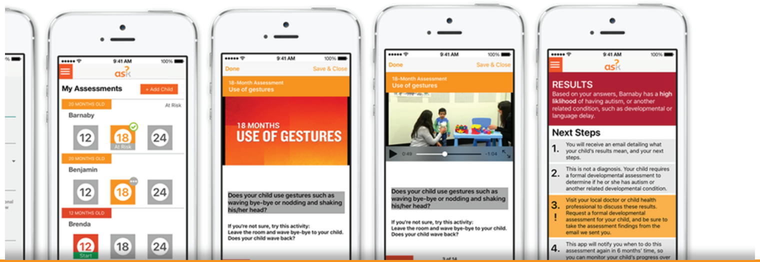

By monitoring your child's development, you're enabling the best outcomes for them. Identifying difficulties in social attention and communication skills early will provide you with the best chance of accessing appropriate services and support for your child.

EARLY IDENTIFICATION

+

EARLY INTERVENTION

=

IMPROVED CHILD AND FAMILY OUTCOMES
